# Benchmarking of germline copy number variant callers from whole genome sequencing data for clinical applications

**DOI:** 10.1093/bioadv/vbaf071

**Published:** 2025-04-10

**Authors:** Francisco M De La Vega, Sean A Irvine, Pavana Anur, Kelly Potts, Lewis Kraft, Raul Torres, Peter Kang, Sean Truong, Yeonghun Lee, Shunhua Han, Vitor Onuchic, James Han

**Affiliations:** Tempus AI, Inc., Chicago, IL 60654, United States; Department of Biomedical Data Sciences, Stanford University School of Medicine, Palo Alto, CA 94304, United States; Real Time Genomics, Ltd., Hamilton 3204, New Zealand; Tempus AI, Inc., Chicago, IL 60654, United States; Tempus AI, Inc., Chicago, IL 60654, United States; Tempus AI, Inc., Chicago, IL 60654, United States; Tempus AI, Inc., Chicago, IL 60654, United States; Tempus AI, Inc., Chicago, IL 60654, United States; llumina, Inc., San Diego, CA 92122, United States; llumina, Inc., San Diego, CA 92122, United States; llumina, Inc., San Diego, CA 92122, United States; llumina, Inc., San Diego, CA 92122, United States; llumina, Inc., San Diego, CA 92122, United States

## Abstract

**Motivation:**

Whole-genome sequencing (WGS) is increasingly preferred for clinical applications due to its comprehensive coverage, effectiveness in detecting copy number variants (CNVs), and declining costs. However, systematic evaluations of WGS CNV callers tailored to germline clinical testing—where high sensitivity and confirmation of reported CNVs are essential—remain necessary. Clinical reporting typically emphasizes CNVs affecting coding regions over precise breakpoint detection. This study benchmarks several short-read WGS CNV detection tools using reference cell lines to inform their clinical use.

**Results:**

While tools vary in sensitivity (7%–83%) and precision (1%–76%), few meet the sensitivity needed for clinical testing. Callers generally perform better for deletions (up to 88% sensitivity) than duplications (up to 47% sensitivity), with poor detection of duplications under 5 kb. Notably, for CNVs in genes commonly included in clinical panels, significantly improved sensitivity and precision were observed when benchmarking against 25 cell lines with known CNVs. DRAGEN v4.2 high-sensitivity CNV calls, post-processed with custom filters, achieved 100% sensitivity and 77% precision on the optimized gene panel after excluding recurring artifacts. This level of performance may support clinical use with orthogonal confirmation of reportable CNVs, pending validation on laboratory-specific samples.

**Availability and implementation:**

The data underlying this article are available in the European Nucleo-tide Archive under project accession PRJEB87628.

## 1 Introduction

The clinical utility of whole-genome sequencing (WGS) lies in its ability to provide a comprehensive and accurate identification of genetic variants, including single nucleotide variants (SNVs), insertions and deletions (indels), mitochondrial variants, and copy number variants (CNVs), all within a streamlined workflow (Cirino *et al.* 2017, Bagnall *et al.* 2018, Neerman *et al.* 2019, Pagnamenta *et al.* 2023, Bagger *et al.* 2024). The cost of short-read WGS has declined significantly over the past decade, driven by advancements in sequencing chemistries, increased read density per flow cell, and the scalability of sequencing instruments (Rodriguez and Krishnan 2023, Eisenstein 2024). SNP arrays have traditionally been the platform of choice for clinical genome-wide CNV detection due to their cost-effectiveness for routine clinical analyses. However, they are limited in their ability to detect small CNVs (<50 kb) and offer imprecise breakpoint characterization (Bowman *et al.* 2025). In contrast, WGS provides base-pair resolution and can detect smaller CNVs missed by arrays, delivering precise breakpoint definitions at the nucleotide level in a single test. The current cost of WGS is nearly equivalent to that of whole-exome sequencing (WES) while offering at least an 8% increase in the diagnostic yield for individuals with genetic diseases (Krusche *et al.* 2019, Eisenstein 2023, 2024, Wojcik *et al.* 2024). Consequently, clinical laboratories are eager to leverage the benefits of this technology and require bioinformatics tools that can deliver accurate variant calls (Marshall *et al.* 2020).

CNVs contribute significantly to genetic diversity and disease (Girirajan *et al.* 2011) yet they pose substantial detection challenges, particularly in targeted sequencing assays (Teo *et al.* 2012). These assays, which have been the mainstay of clinical sequencing, typically detect CNVs based solely on subtle changes in sequencing coverage depth across exon footprints (Kadalayil *et al.* 2015). Notably, the sensitivity for detecting single-exon deletions or duplications—a critical task in clinical testing—has been reported to be around 50% at typical sequencing depths for WES (80–120×) (Babadi *et al.* 2023). In contrast, WGS facilitates the detection of entire CNV spans, often including intronic sequences not captured in targeted panels. This enables the identification of CNV breakpoints and provides an additional layer of data beyond depth measurements, allowing for more accurate variant pinpointing (Medvedev *et al.* 2009).

Although CNV detection from WGS data may appear to be a resolved issue, evidence to support this claim remains limited. Recent advancements in CNV callers have predominantly focused on the identification of these variants from targeted sequencing data (Kadalayil *et al.* 2015, Moreno-Cabrera *et al.* 2020, Gordeeva *et al.* 2021). Emerging methods, including hardware accelerated algorithms (Behera *et al.* 2024) and deep learning (Popic *et al.* 2023, Mandiracioglu *et al.* 2024) promise enhanced accuracy; however, comprehensive benchmarking of these approaches is lacking. Moreover, tools for short-read WGS CNV detection must be rigorously evaluated for clinical applications, where orthogonal confirmation of CNVs is typically required (Crooks *et al.* 2023). In this context, the emphasis shifts to achieving high sensitivity over specificity or precision, contrasting with the priorities of research applications.

In this study, we aimed to evaluate CNV calling tools designed for short-read, PCR-free WGS data. We used cell lines with known CNVs to assess their suitability for clinical gene-panel reporting from 50× WGS data. The accuracy of several CNV callers was compared using the Genome-in-a-Bottle (GIAB) consortium HG002 reference cell line (Zook *et al.* 2019) and a panel of 25 additional cell lines, each with documented CNVs in a selection of clinically relevant genes.

## 2 Materials and methods

### 2.1 Cell lines

For whole-genome benchmarking, we utilized the HG002 cell line characterized by the Genome-in-a-Bottle (GIAB) Consortium (Zook *et al.* 2019). Additionally, we selected 25 cell lines from the Coriell Institute catalog with reported CNVs (Supplementary Table S1). These cell lines include CNVs overlapping genes in a panel comprising 89 hereditary cancer genes, 79 cardiometabolic disease genes, and 20 rare genetic disease genes, a total of 184 unique genes after removing redundancies (Supplementary Table S1).

### 2.2 Sample preparation and sequencing

PCR-free WGS libraries from the DNA of Coriell Institute’s cell lines were sequenced to a mean depth of 50× using paired-end 2 × 150 bp reads on the Illumina NovaSeq 6000 system. The reads were mapped to the human reference genome GRCh37 using the DRAGEN Secondary Analysis Platform (Behera *et al.* 2024).

### 2.3 CNV detection tools evaluated

We evaluated the following CNV calling tools for short-read WGS: Delly v1.6 (Rausch *et al.* 2012), CNVnator v0.4.1(Abyzov *et al.* 2011), Lumpy v0.2.13 (Layer *et al.* 2014), Parliament2 (Zarate *et al.* 2020), Cue (cue.v2.pt model) (Popic *et al.* 2023), and the DRAGEN 4.2 integrated CNV-structural variation (SV) caller (DRAGEN Secondary Analysis Platform, Illumina, Inc.) (Behera *et al.* 2024). We tested DRAGEN in two modes: default parameters and high-sensitivity mode (enabled with the -sv-cnv-enable-high-sensitivity-mode=true option, hereafter referred to as DRAGEN HS). All tools used default settings recommended by their developers and started from the same BAM alignments against GRCh37 generated with the DRAGEN multi-genome (graph) aligner (Behera *et al.* 2024).

### 2.4 Benchmarking data analysis

Given the clinical focus, we evaluated CNV calls based on their potential to disrupt protein structure. True positives were defined as events overlapping at least 1 bp of coding exons from canonical transcripts (plus 15 bp of intronic sequence to capture splice junctions) and matching the dosage direction in the CNV truth set. Events not meeting these criteria were classified as false positives. To avoid double counting, events spanning multiple exons were adjusted. Sensitivity and precision were calculated using the Global Alliance for Genomes and Health (GA4GH) Benchmarking Team definitions (Krusche *et al.* 2019).

For whole-genome analysis, the HG002 v0.6 truth set from GIAB on GRCh37 (hs37d5) was used as the reference (Zook *et al.* 2019). Since the GiaB truth set represents duplications as insertions, we compared the sequence of the insertions to the reference genome, requiring a minimum of 95% identity, and allowing up to a 2 bp slop between repeats to identify duplications. Confirmed duplications were converted to DUP VCF records; others were excluded. Analysis focused on events ranging from 500 bp to 10 Mb. The original truth set included 13 deletions and 4 duplications, overlapping 45 and 8 exons of canonical transcripts, respectively.

Given the relatively few exon overlaps observed, synthetic gene models were added to the canonical transcript exons to enhance statistical analysis. Gene models from *BRCA1*, *BRCA2*, *CHEK2*, *PLP1*, and *GAA* were used as templates to construct synthetic genes within high-confidence regions of HG002 as specified in the HG002_SVs_Tier1_v0.6.bed file provided alongside the GiaB truth set for this cell line. Synthetic genes were placed avoiding overlapping real human genes, with a minimum 100 kb gap between consecutive genes. We added a few gene models directly on top of the duplications in the SV truth set to increase the number of exon overlaps with this sparse variant type. This approach yielded 5213 synthetic genes comprising 79 397 exons, adding 47 deletions and 6 duplications overlapping 94 and 19 exons, respectively (c.f. Supplementary File S1).

For the collection of 25 cell lines, we used the CNV annotations described on the Coriell Institute website as the initial truth set. Since the reported alterations for cell lines in the Coriell catalog are sometimes incomplete, we further curated the truth set by visually inspecting the alignments of any putative false positives within the gene panel. Variants deemed convincing based on coverage graphs were added to the truth set (Supplementary Table S1 and Fig. S5). Seven cell lines in the study harbored CNV events in genes requiring a specialized caller due to extensive paralogy (e.g. *CYP2D6*, *GBA*, and *PMS2*; not listed in the Supplementary Table S1). CNV calls across these genes were excluded from evaluation.

### 2.5 Custom artifact filter for DRAGEN HS

We designed a custom filtering scheme for DRAGEN HS calls to maximize sensitivity for the gene panel while minimizing false positives. The filtering was implemented using the RTG vcffilter tool with a custom JavaScript (Cleary *et al.* 2015). First, records smaller than 500 bases (where DRAGEN HS generates numerous false positives) and larger than 10 Mb (where recurrent breakpoint-based artifacts spanning centromeres or telomeres were observed) were excluded. Calls based solely on junction reads exceeding 1 Mb (denoted in the VCF as SVCLAIM=J in the INFO field) were also discarded due to artifact prevalence, as true calls in this range generally exhibit depth support. Additionally, calls overlapping centromeres and telomeres—a common source of breakpoint false positives—were removed by cross-referencing the UCSC genome browser “gap” track BED file. Finally, calls with a reciprocal overlap of ≥90% with recurrent artifacts identified across cell lines were excluded. Due to technical constraints of the RTG vcffilter tool, this step was implemented using two BED files: one specifying the minimum overlap and the other the maximum. Filtered calls are hereafter referred to as DRAGEN HS-F. The custom JavaScript script for use with the RTG vcffilter tool, BED files, and a flow diagram of the filtering process are provided in Supplementary File S1 and Fig. S1.

## 3 Results

### 3.1 Whole-genome benchmarking

We evaluated CNV callers at the genome level using WGS data from the HG002 cell line, which has been extensively characterized by the GIAB consortium. A genome-wide truth set for CNVs and structural variants is available for this cell line in GRCh37 (Zook *et al.* 2019). The GIAB v0.6 truth set included 13 deletions and 4 duplications, overlapping 45 and 8 exons of GRCh37 canonical transcripts, respectively. This cell line, derived from a healthy individual, does not harbor pathogenic CNVs in major disease genes. To improve our statistical analysis, we also simulated gene models on top of other variants in the truth set to increase the number of overlaps evaluated. This simulation added 47 deletions and 6 duplications, overlapping 94 and 19 exons, respectively.

We included five commonly used open-source WGS CNV callers in our evaluation: Delly (Rausch *et al.* 2012), CNVnator (Abyzov *et al.* 2011), Lumpy (Layer *et al.* 2014), and Parliament2 (Zarate *et al.* 2020). Additionally, we evaluated two newer WGS CNV callers: Cue, a deep-learning-based caller (Popic *et al.* 2023) and the DRAGEN 4.2 CNV-SV integrated caller, part of the hardware-accelerated DRAGEN Secondary Analysis Platform (Behera *et al.* 2024). DRAGEN was tested in both standard and high-sensitivity mode (HS), which prioritizes sensitivity over precision. We further evaluated the DRAGEN HS calls after applying a custom filtering process (referred to as DRAGEN HS-F; see Section 2).

**Figure 1.**
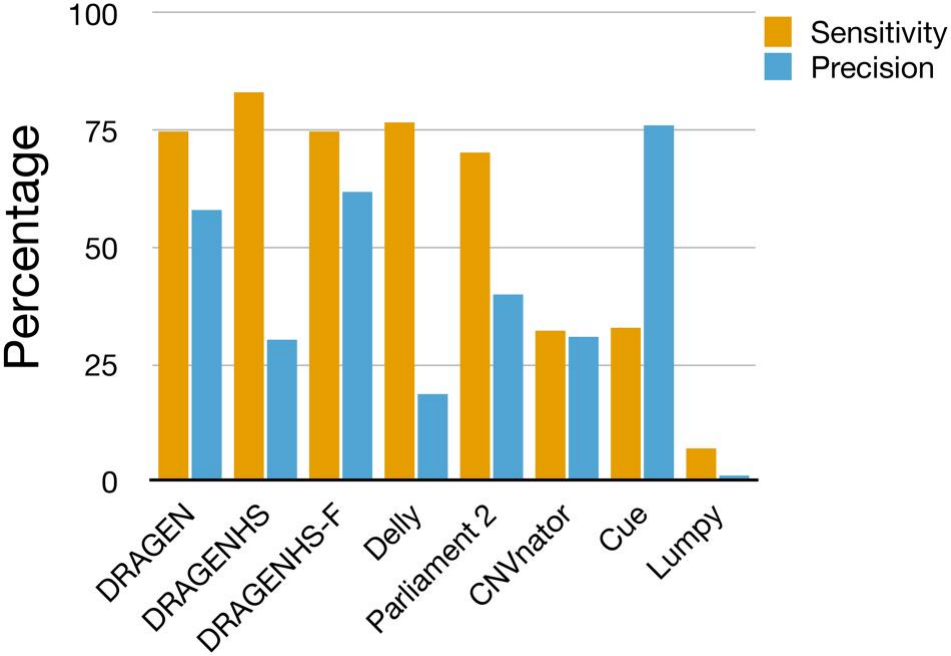
Genome-wide performance of CNV callers. DRAGEN v4.2 CNV caller achieved the best balance of sensitivity and precision. In high-sensitivity mode (DRAGEN HS), it demonstrated the highest sensitivity but with reduced precision. Cue achieved the highest precision but had lower sensitivity, partly due to its inability to detect events smaller than 5 kb. Custom filters applied to DRAGEN HS (referred to as DRAGEN HS-F) improved precision with only a slight reduction in sensitivity.

Sensitivity and precision were evaluated for events overlapping exons of both canonical and synthetic transcripts. The truth set included a total of 60 deletions and 10 duplications, each overlapping one or more exons. Our results (Fig. 1) showed that the maximum combined sensitivity (for deletions and duplications) was achieved by DRAGEN HS (83%), while the maximum precision was observed with Cue (76%). Delly exhibited high sensitivity (77%) but the lowest precision. Despite its high precision, Cue had relatively low sensitivity (33%), primarily due to its inability to detect events smaller than 5 kb, which was the lower limit of its training model. Lumpy demonstrated low sensitivity and precision in all tests. DRAGEN HS achieved the highest sensitivity (83%) but at the cost of lower precision (30%). After applying custom filters (DRAGEN HS-F), precision improved significantly with only a small reduction in sensitivity (75%), providing the best balance between sensitivity and precision.

**Figure 2.**
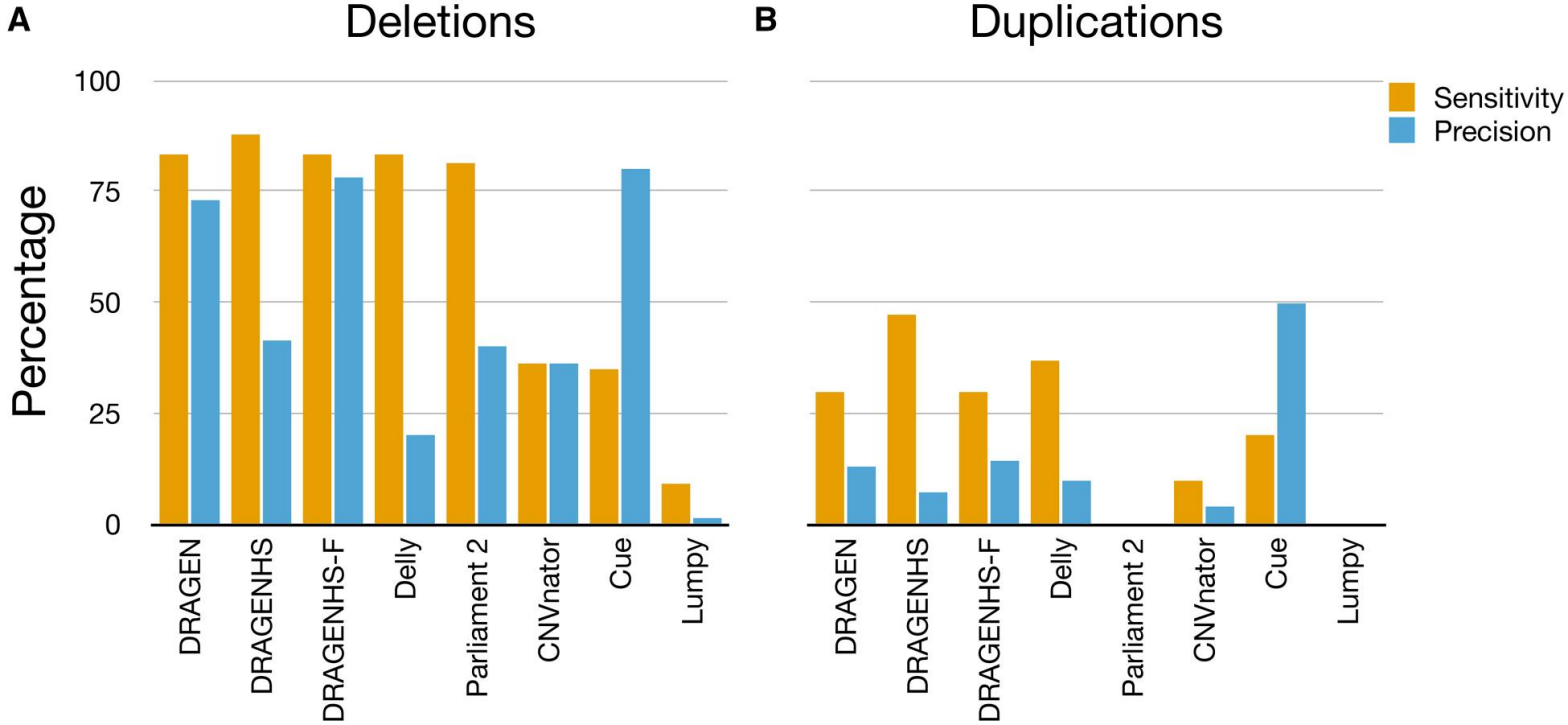
Genome-wide performance of the CNV callers stratified by CNV type. The performance for deletions followed trends observed in the overall metrics in Fig. 1. Sensitivity for duplications was significantly lower across all callers, with DRAGEN HS achieving the highest sensitivity and Cue achieving the highest precision.

Next, we stratified the performance of the callers by deletions and duplications (Fig. 2). Performance for deletions followed trends observed in overall metrics (Fig. 1). However, sensitivity for duplications was significantly lower across all CNV callers. DRAGEN HS exhibited the highest sensitivity for duplications (47%), while Cue had the highest precision (50%).

**Figure 3.**
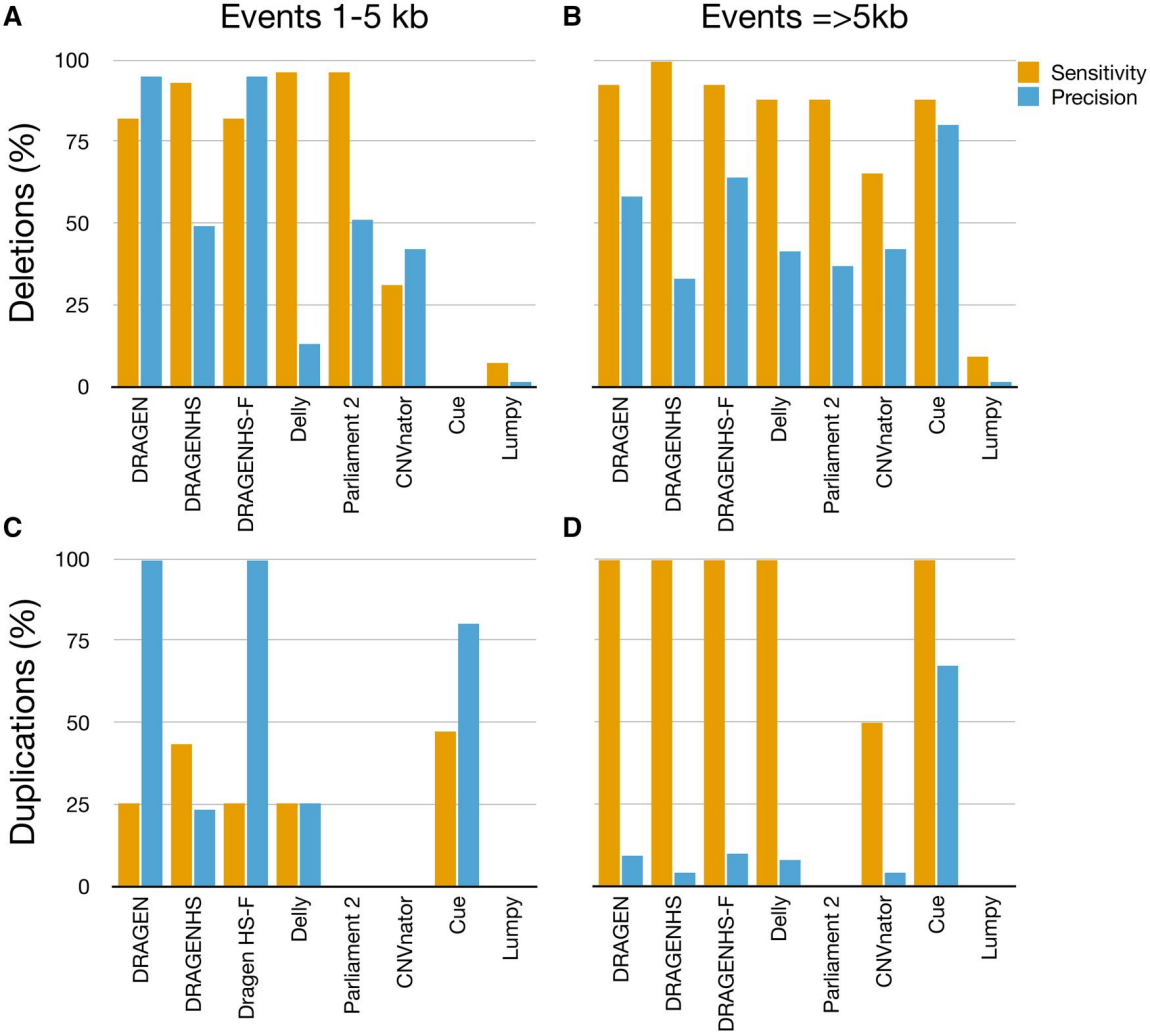
Genome-wide performance of the CNV Callers stratified by event length. Results are categorized by event size, either 1–5 kb or larger than 5 kb. All callers exhibited reduced sensitivity for smaller events, with some unable to detect events in the 1–5 kb range. Sensitivity for duplications.

Finally, we evaluated combined performance (deletions and duplications) by event length (Fig. 3): 1–5 kb and ≥5 kb (up to 100 kb). All callers demonstrated lower sensitivity for events of 1–5 kb, with CNVnator and Cue unable to detect events in this range. The lack of calls in the 1–5 kb range for Cue was expected, as its current model was not trained to detect events of this length. DRAGEN achieved the highest precision for small duplications. Surprisingly, while many callers showed high sensitivity for duplications larger than 5 kb, their precision remained low except for Cue. Results for additional CNV length range stratification showing similar trends are presented in Supplementary Table S3, though metrics could not be calculated in some cases due to the limited number of true positive events in certain strata (e.g. 5–10 kb).

### 3.2 Benchmarking for virtual gene-panels from WGS data

We expanded our evaluation to include 25 cell lines from the Coriell Institute catalog annotated with CNVs in exons of a virtual panel of 184 genes commonly tested for hereditary cancer, cardiovascular disease, and rare genetic disorders (Supplementary Table S2). Since the rest of the genome in these cell lines has not been comprehensively characterized, the evaluation was limited to the exons of the virtual panel. Putative false positives identified during evaluations were inspected using running depth coverage patterns across the genome to determine if these were real events. This step was essential, as cell lines often harbor events not listed in catalog metadata. Ultimately, several non-listed events were included in the truth set.

**Figure 4.**
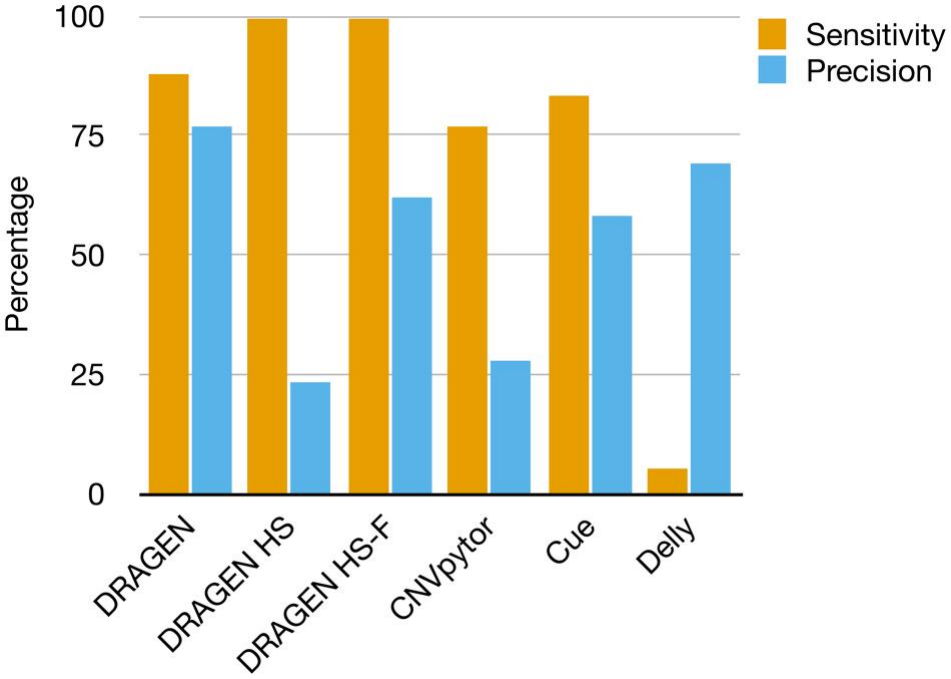
Gene-panel level performance. DRAGEN v4.2 CNV caller demonstrated the best balance between sensitivity and precision in the gene-panel use case. When run in high-sensitivity mode (DRAGEN HS), it achieved the highest sensitivity, albeit with reduced precision. The application of custom filters (DRAGEN HS-F) improved precision without sacrificing sensitivity. In contrast, other callers in the study showed lower sensitivity and precision.

In the 184-gene virtual panel, DRAGEN HS exhibited the highest sensitivity (100%), while DRAGEN with standard settings achieved the highest precision (77%) (Fig. 4). Notably, applying custom filters to DRAGEN HS improved precision from 23% to 62% without reducing sensitivity. Other callers demonstrated lower sensitivity and precision overall, although Delly showed higher precision in this evaluation than in the WGS analysis. Based on the promising performance of DRAGEN HS, we further analyzed its performance stratified by CNV event size, using both the number of exons spanned and event length (Table 1). Sensitivity remained at 100% across all strata, while precision for DRAGEN HS-F was 100% for events smaller than 1 kb or overlapping a single exon but decreased to 68% for events spanning five or more exons.

**Table 1.** Precision of CNV detection in virtual panels stratified by number of exons affected or event length for different DRAGEN modalities.

Exons spanned			CNV length		
No. of exons	Precision HS (%)	Precision HS-F (%)	Length (kb)	Precision HS (%)	Precision HS-F (%)
1	8	100	0.5–1	100	100
2–5	10	81	1–10	30	89
>5	1	68	>10	2	74

**Figure 5.**
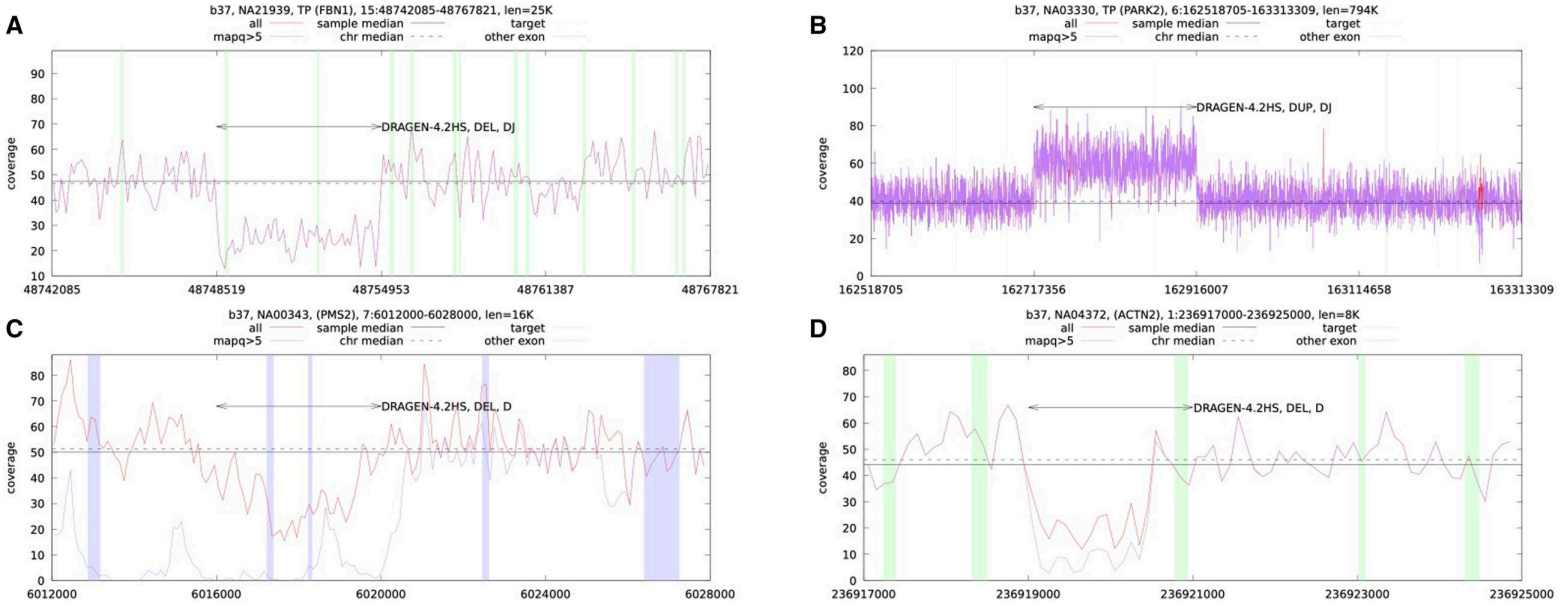
Examples of true positive and false positive CNV calls for DRAGEN HS-F. Coverage graphs (100 bp bins) show DRAGEN CNV calls marked with arrowheads. “D” denotes calls supported by depth analysis, and “J” denotes calls supported by junction reads (breakpoints). Shaded green vertical areas represent exons of canonical transcripts from the gene panel, while other regions are shaded in blue. (A) True positive (TP) deletion (DEL) in *FBN1*. (B) TP duplication (DUP) in *PARK2*. (C) False positive (FP) deletion in the *PMS2* gene caused by mappability issues in regions paralogous to the *PMS2CL* pseudogene (evidenced by a drop in MAPQ >5 coverage line). (D) TP deletion in *ACTN2* where an overextended right breakpoint results in a FP exon overlap.

To gain insights into failure modes, we examined coverage patterns for genomic regions with true and false positives from DRAGEN HS-F calls. Figure 5A and B show examples of true positive deletions and duplications supported by both depth and junction breakpoints. Figure 5C illustrates a false positive deletion caused by a mappability issue in regions paralogous to the *PMS2CL* pseudogene. Figure 5D depicts a false positive deletion in *ACTN2*, which is a real deletion; however, the overextension of the 3′-end breakpoint results in an exon overlap classified as a false positive. False positives were most commonly observed for duplications ranging from 1–10 kb in length.

## 4 Discussion

Given the critical role that CNV play in genetic diseases (Girirajan *et al.* 2011) the performance of CNV detection tools using next-generation sequencing (NGS) data can significantly impact the yield and accuracy of clinical genetic tests. Improving sensitivity for CNV detection in targeted sequencing panels—the workhorse of clinical genetic testing to date—has been challenging, particularly for single-exon events (Babadi *et al.* 2023). This difficulty stems from the limited data available to distinguish copy number status from deletions or amplifications based solely on sequencing coverage depth, especially given the short length of typical exons (∼120bp) (Teo *et al.* 2012, Kadalayil *et al.* 2015). Some of this sensitivity loss can be mitigated by increasing sequencing depth (e.g. from ∼200× to ≥500×) at the expense of increased assay cost. This situation is poised to improve with the decreasing costs of WGS and its growing adoption in clinical testing (Eisenstein 2023, 2024). Unlike targeted gene panels or WES, which only capture and sequence coding exon regions, WGS provides data across the entire span of a CNV, often including non-coding regions, and its breakpoints (Medvedev *et al.* 2009). This additional information can be incorporated into CNV algorithms to improve the sensitivity of variant detection, even if the event only overlaps a single exon. Numerous bioinformatics tools have been developed over the past decade for this purpose (Kadalayil *et al.* 2015, Moreno-Cabrera *et al.* 2020, Gordeeva *et al.* 2021). However, comprehensive benchmarking focused on clinical genetic testing needs remains limited, particularly for commercial tools widely used in clinical laboratories.

To date, only two benchmarks of short-read WGS CNV callers have been published. Whitford *et al.* (2019) reported a comparison of four CNV callers, limited to deletions and using an older truth set from the GiaB pilot sample (NA12878/HG001). Gabrielaite *et al.* (2021) analyzed 11 CNV callers, including one commercial tool, using 38 samples sequenced as WGS with corresponding SNP-array CNV calls. Both studies had significant limitations in truth sets and tool selection. In this study, we aimed to address these gaps by benchmarking commonly used WGS CNV callers against a more recent and comprehensive truth set. We also included two newer callers that implement innovative approaches: Cue, which uses a novel deep learning paradigm for structural variant identification (Popic *et al.* 2023), and the DRAGEN 4.2 integrated CNV-SV caller, part of an FPGA-accelerated bioinformatics software suite that consolidates two independent approaches for CNV calling, namely, depth and breakpoint-based (Behera *et al.* 2024).

A major difference with prior benchmarks, is our focus on the needs of clinical genetic testing, where the relevance of CNVs is determined by their ability to disrupt coding regions and impact the resulting protein sequence. Accordingly, we evaluated the callers based solely on their overlap with coding regions, rather than assessing the accuracy of breakpoint identification or event length overlap. Critically, our evaluation prioritized sensitivity, as clinical gene panel testing demands over 99% sensitivity. This high threshold is essential because missing a clinically relevant variant in medical genetic testing is unacceptable, even at the expense of lower precision (Crooks *et al.* 2023). Lower precision is typically addressed through orthogonal validation using secondary technologies (e.g. microarrays, RT-PCR, or long-read sequencing) to achieve a test-level precision of over 99% (Crooks *et al.* 2023). As long as the rates of true positive CNVs and false positives remain within single digits, the cost of validation amortized across all cases is acceptable.

We used the GiaB HG002 cell line for genome-wide evaluation, which serves as a genomic standard developed by the US National Institute of Standards and Technology, and is being characterized as part of the Genome-in-a-Bottle consortium (Zook *et al.* 2019). While the development of a comprehensive truth set for structural variants and CNVs in HG002 is a work in progress, we relied on the interim v0.6 release (Zook *et al.* 2020). Additionally, we evaluated cell lines known to harbor CNVs in genes frequently included in clinical panels for hereditary cancer, cardiovascular disease, and rare genetic disorders (Cirino *et al.* 2017, Bagger *et al.* 2024). Performance was solely based on the coding regions of the genes in the aggregated panels encompassing 184 genes (Supplementary Table S2). Results were further stratified by event type (deletion or duplication) and event size/length to identify tool-specific strengths and weaknesses.

The CNV detection tools benchmarked in this study use diverse algorithms that directly influence their performance. Depth-based methods, such as CNVnator (Abyzov *et al.* 2011, Suvakov *et al.* 2021) and DRAGEN CNV (Behera *et al.* 2024), detect copy number deviations by binning the genome and comparing sequencing depths to reference-based models (e.g. accounting for GC content and mappability). These tools are robust for detecting large CNVs but may struggle with breakpoint resolution and smaller events. Breakpoint-based methods, like Delly (Rausch *et al.* 2012), Lumpy (Layer *et al.* 2014), and DRAGEN SV analyze split-read alignments to identify breakpoints. Lumpy also detects unexpected paired-end read distances. Hybrid approaches integrate multiple algorithms to improve overall performance. For example, Parliament2 combines predictions from six independent tools (Zarate *et al.* 2020) while the DRAGEN integrated CNV-SV caller integrates depth- and breakpoint-based analyses (Behera *et al.* 2024). These differences in focus and methodology result in varying strengths and weaknesses across tools. For instance, while depth-based tools are more reliable for large CNVs, split-read and deep-learning methods excel in breakpoint detection but may yield more false positives. Ensemble approaches, though robust, often introduce additional computational complexity and optimization challenges. Deep learning-based methods, such as Cue (Popic *et al.* 2023), analyze alignment-derived data by machine learning but require extensive training datasets, which in our study limited its ability to detect variants smaller than 5 kb in this study. Moreover, developers of CNV calling algorithms typically strive to maximize the F1 score, aiming to balance true positives (TPs) and false positives (FPs). Importantly, this optimization may not be suitable for all applications, as different trade-offs may be more desirable in some situations, as highlighted in this study. Flexibility in parameterization is, therefore, a crucial feature for CNV callers intended for clinical use.

In our results, DRAGEN HS emerged as the most sensitive tool, achieving 100% sensitivity, a critical requirement for clinical applications. However, its precision at initial settings was lower, a common trade-off in high-sensitivity tools. Custom filters (DRAGEN HS-F) significantly improved precision without sacrificing sensitivity, demonstrating the potential of optimized bioinformatics pipelines to balance trade-offs based on the specific requirements of the application.

Limitations of our study include the potential overfitting of the DRAGEN HS filtering scheme, as excluded recurrent artifacts were specific to the evaluated genes. Nonetheless, the principles of this scheme could be readily adapted to other settings or gene panels. Additionally, our evaluation was limited to events ranging from 1 kb to 10 Mb, consistent with our focus on screening clinical gene panels, where large aberrations are less common. Performance for events between 250 bp and 1 kb is likely suboptimal, and the detection of large genomic events associated with rare diseases requires further investigation using appropriate samples. Furthermore, we were unable to assess the performance of CNV callers on well-known challenging, medically relevant genes (Wagner *et al.* 2022). due to a lack of samples harboring pathogenic CNVs in these regions. Finally, bespoke callers have been developed to detect CNVs in genes with regions of high paralogy, such as *CYP2D6*, *GBA*, *SMN1*, *SMN2*, *LPA1*, and *PMS2*. However, their evaluation was beyond the scope of the current study.

## 5 Conclusions

This study highlights the critical need for continuous benchmarking and refinement of CNV detection tools to meet the demands of clinical diagnostics. DRAGEN v4.2 HS-F, with its adjustable balance between sensitivity and precision, demonstrates strong potential for integrating WGS into clinical diagnostic pipelines. Further advancements in CNV detection methods are essential to enhance analytical accuracy while reducing both costs and reliance on orthogonal validation of WGS results.

## Supplementary Material

vbaf071_Supplementary_Data

## Data Availability

The data underlying this article are available in the European Nucleotide Archive under project accession PRJEB87628.
