# Supplementary figures and images for: Benchmarking of germline copy number variant callers from whole genome sequencing data for clinical applications
Source: Bioinform Adv. 2025 Apr 10;5(1):vbaf071. doi: 10.1093/bioadv/vbaf071 (PMC12005901; doi:10.1093/bioadv/vbaf071)

**Supplemental Figure 1.** Workflow diagram of the filtering scheme for DRAGEN HS CNV calls.

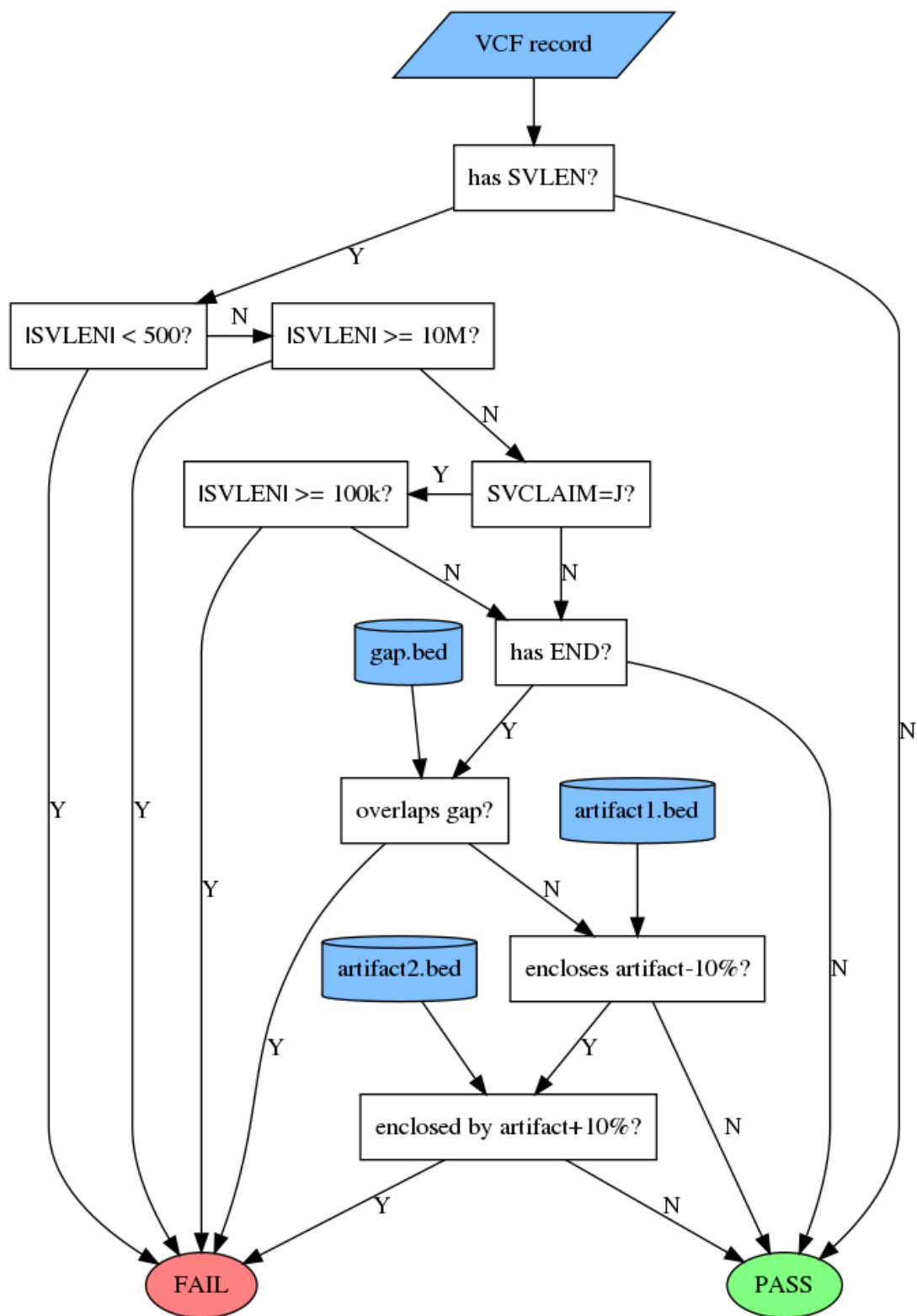

Supplement: vbaf071_Supplementary_Data [file vbaf071_supplementary_data.zip › Supplemental_Figure_1.pdf]
